# Emerging Biomarkers for Prediction and Early Diagnosis of Necrotizing Enterocolitis in the Era of Metabolomics and Proteomics

**DOI:** 10.3389/fped.2020.602255

**Published:** 2020-12-08

**Authors:** Eleni Agakidou, Charalampos Agakidis, Helen Gika, Kosmas Sarafidis

**Affiliations:** ^1^1st Department of Neonatology, Faculty of Medicine, Ippokration General Hospital, Aristotle University of Thessaloniki, Thessaloniki, Greece; ^2^1st Department of Pediatrics, Faculty of Medicine, Ippokration General Hospital, Aristotle University of Thessaloniki, Thessaloniki, Greece; ^3^Laboratory of Forensic Medicine and Toxicology, Faculty of Medicine, Aristotle University of Thessaloniki, Thessaloniki, Greece; ^4^BIOMIC_AUTH, Bioanalysis and Omics Laboratory, Centre for Interdisciplinary Research and Innovation, CIRI-AUTH B1.4, Aristotle University of Thessaloniki, Thessaloniki, Greece

**Keywords:** neonates, preterm infants, necrotizing enterocolitis, proteomics, metabolomics, medical NEC, LC-MS/MS, NMR spectroscopy 3

## Abstract

Necrotizing Enterocolitis (NEC) is a catastrophic disease affecting predominantly premature infants and is characterized by high mortality and serious long-term consequences. Traditionally, diagnosis of NEC is based on clinical and radiological findings, which, however, are non-specific for NEC, thus confusing differential diagnosis of other conditions such as neonatal sepsis and spontaneous intestinal perforation. In addition, by the time clinical and radiological findings become apparent, NEC has already progressed to an advanced stage. During the last three decades, a lot of research has focused on the discovery of biomarkers, which could accurately predict and make an early diagnosis of NEC. Biomarkers used thus far in clinical practice include acute phase proteins, inflammation mediators, and molecules involved in the immune response. However, none has been proven accurate enough to predict and make an early diagnosis of NEC or discriminate clinical from surgical NEC or other non-NEC gastrointestinal diseases. Complexity of mechanisms involved in NEC pathogenesis, which remains largely poorly elucidated, could partly explain the unsatisfactory diagnostic performance of the existing NEC biomarkers. More recently applied technics can provide important insight into the pathophysiological mechanisms underlying NEC but can also aid the detection of potentially predictive, early diagnostic, and prognostic biomarkers. Progress in omics technology has allowed for the simultaneous measurement of a large number of proteins, metabolic products, lipids, and genes, using serum/plasma, urine, feces, tissues, and other biological specimens. This review is an update of current data on emerging NEC biomarkers detected using proteomics and metabolomics, further discussing limitations and future perspectives in prediction and early diagnosis of NEC.

## Introduction

Necrotizing enterocolitis (NEC) is a disastrous disease of neonates that mainly affects very low birth weight infants (VLBWI). Despite the progress made in the management of preterm infants, the incidence of NEC has not declined over the last four decades (1–5). In a prospective registry of 34,636 infants with a gestational age (GA) ranging from 22 to 28 weeks, born at 26 network centers from 1993 through 2012, the incidence of NEC ranged between 7 and 13% without a clear upward or downward trend over the years (4). Recent systematic reviews on NEC incidence found in high income countries reported considerable differences among studies with an NEC rate varying from 2 to 7% in preterm infants with GA of lower than 32 weeks' gestation and 5–22% in those with birth weight (BW) of < 1,000 grams (2). NEC is a leading cause of death in VLBWI with a mortality rate ranging from 16 to 42% depending on BW/GA (6, 7). Moreover, NEC survivors are at high risk of severe long-term complications such as short bowel syndrome and neurodevelopmental sequelae, which are associated with low quality of life and increased long-term medical expenses.

Almost 60 years after the identification of NEC as a distinct entity in preterm infants, certain disease issues have not been fully clarified. An important obstacle to NEC understanding is the lack of a reliable definition, including those characteristics that could differentiate NEC from non-NEC gastrointestinal diseases of the neonate, also referred to as NEC-like diseases (8). The dominant view is that the term “necrotizing enterocolitis” does not refer to a single entity but rather to a spectra of neonatal gastrointestinal diseases with different pathophysiology and clinical, radiology, and pathology characteristics. In this context, certain entities that are generally considered as NEC are in fact imitators of the preterm infant's NEC, such as the isolated intestinal perforation, intestinal ischemia-hypoxia secondary to perinatal asphyxia and congenital heart disease, enterocolitis associated with Hirschsprung's disease, food protein-induced enterocolitis syndrome, and viral enteritis (8–10). Finally, differentiating medical from surgical NEC has a major impact on decision making (11).

Herein, after a brief reference to the pathophysiology of NEC and current diagnostic means, we provide an overview of experimental and clinical data regarding the discovery and evaluation of candidate biomarkers for prediction, diagnosis, and prognosis of NEC utilizing metabolomic and proteomic analyses.

## Pathophysiology and Current Diagnosis of Necrotizing Enterocolitis

### Pathophysiology and Factors Affecting NEC Development

The pathophysiology of NEC is multifactorial and has not as of yet been fully clarified. Prematurity, formula feeding, and intestinal dysbiosis are the main risk factors, while the genetic predisposition as well as certain prenatal and postnatal factors increase the risk of NEC (Table 1) (12–21).

**Table 1:** Prenatal and postnatal factors involved in NEC pathophysiology (references 12–21).

**A. Main causative factors**
• Prematurity • Formula feeding • Intestinal dysbiosis
**B. Prenatal factors associated with increased NEC risk**
• Genetic predisposition • Chorioamnionitis • Intrauterine growth restriction • Placenta abruption • Preeclampsia • Prenatal/perinatal asphyxia • Prenatal antibiotics • Cesarean section • Lack of prenatal steroids • Prolonged rupture of membranes
**C. Postnatal factors associated with increased NEC risk**
• Hypoxia • Congenital heart disease • Anemia • Decreased intestinal perfusion • Patent ductus arteriosus • Blood transfusions (?) • Fortification of human milk with cow's milk - derived fortifier • Medications □ Excessive antibiotic use □ Indomethacin □ Acid suppressive H2 blockers

Intestinal immaturity of preterm infants is key in NEC development and severity as indicated by the strong, inverse relationship of GA with NEC incidence and associated mortality (22). The preterm infants' intestine is characterized by a vulnerable mucosal barrier, immature immune system, dysregulated inflammatory response, altered microbiome composition and diversity, and immature motility and digestion (23–27). Intestinal barrier dysfunction allows bacteria translocation that exaggerates the inflammation cascade, leading to mucosal inflammatory infiltration, upregulation of inflammatory mediators, including cytokines, chemokines, and adhesion molecules, coagulation activation, and ischemia, which further induces intestinal injury and necrosis (18, 25, 28, 29).

The intestinal microbiome plays a regulatory role in intestinal immune defense. Prevalence of commensal bacteria protects against bacterial translocation and exaggeration of local inflammation, while proteobacteria predispose to inflammation and pathogen invasion, increasing the risk of NEC (9, 17). Existing evidence supports a dominant role for microbial dysbiosis in NEC pathogenesis (18, 19, 21, 30–32), while experimental studies showed that germ-free animals do not develop NEC (26). Clinical studies demonstrated significant differences in gut microbiota composition between low birth weight and normal birth weight infants as well as between infants with and without NEC (33, 34). Moreover, a systematic review suggested that alterations in intestinal microbiota by means of decreased firmicutes and Bacteroidetes and increased proteobacteria precede the onset of NEC (35). In this context, feeding preterm infants with maternal milk, which favors intestinal colonization by commensal bacteria, is best for NEC prevention (18). On the other hand, practices favoring intestinal colonization by pathogenic bacteria, such as formula feeding and birth by caesarian section, are associated with increased risk of NEC (17, 18, 36, 37). Data on the effect of antibiotics on NEC occurrence are contradictory. The current view that antibiotics increase the risk of NEC is based on clinical studies showing that intrapartum and early, prolonged antibiotic treatment is associated with altered intestinal microbiome composition and increased incidence of NEC and/or death (38–40). Interestingly, experimental studies in preterm piglets showed that short antibiotic treatment within the first 4 to 5 days of life depressed the bacterial microbiome and reduced the incidence of NEC (41, 42). However, as further noted by the authors in the latter metabolomic study, reduced availability of certain proteins associated with gut microbiome function may adversely affect the development and functions of the gut and central nervous system (42).

Less is known regarding the importance of genetic predisposition in NEC development. Epidemiological studies showed differences in NEC prevalence among populations of different ethnic origin, while a study of twins provided evidence of a familiar predisposition to NEC (3, 43, 44). Genetic studies reported variations of genes and RNAs involved in NEC pathophysiology, predominantly inflammatory mediators, indicating genetic predisposition to NEC (43, 45–48). A recent review by Cuna et al. refers to the available data on genetic predisposition to NEC (43).

### Current Diagnosis of NEC

#### Clinical and Radiological Diagnosis

It is current clinical practice to diagnose NEC based on the clinical, radiological, and hematological findings constituting Bell's criteria, as per a recent review (49). However, Bell's criteria are not specific for NEC and cannot differentiate between NEC and non-NEC diseases (8, 10). Moreover, Bell's stage I does not always progress to stages II and III, and many neonatologists thus considered it a pre-NEC condition (9, 10). Specifically, clinical findings of NEC include increased gastric residuals, feeding intolerance, bloody stool, and abdominal distension; these may progress to generalized hypotonia, lethargy, and cardio-respiratory failure, which can also be features of other neonatal conditions, including sepsis, and viral intestinal infections (9, 10).

Early radiological findings of dilated bowel loops and a thickened bowel wall may also be caused by either obstruction or sepsis without NEC (9, 10, 50). Pneumatosis intestinalis and signs of intestinal perforation develop later in the course of NEC while they may also appear in non-NEC intestinal diseases. These data combined with the reported high inter-observer variability of radiological signs of NEC suggest that radiological findings cannot accurately predict and diagnose NEC (51).

Bowel ultrasound (US) and doppler ultrasonography can be used complementary to abdominal radiology in NEC diagnosis (50, 52). The US is able to assess in real time intestinal wall peristalsis and thickness, intramural and porta venous gas, free abdominal fluid, bowel wall perfusion and superior mesenteric artery flow. The reported diagnostic value of abdominal US for NEC varies depending on the kind and number of US features assessed (53). A recent systematic review and metanalysis of 11 studies including 748 infants found that several bowel ultrasound findings were significant predictors of progression to surgical NEC and death (54). Two recent studies exploring the utility of the superior mesenteric artery doppler ultrasonography in NEC diagnosis reported that decreased systolic and diastolic velocities and increased resistive index were associated with NEC development and could differentiate between septic preterm infants with NEC and those without NEC (55, 56). These findings support the involvement of ischemia in the intestinal damage and indicate the potential utility of upper mesenteric doppler ultrasonography in prediction and early diagnosis of NEC.

Near-infrared spectroscopy has been evaluated as a candidate tool for NEC diagnosis (57). Schat et al. assessed the potential use of cerebral and splanchnic fractional tissue oxygen extraction, as a marker for intestinal underperfusion in NEC, using plasma levels of the I-FABP as an indicator of intestinal damage (58). They found that both cerebral and splanchnic fractional tissue oxygen extraction were strongly inversely associated with plasma levels of the I-FABP. The authors concluded that NEC-associated intestinal injury is accompanied by intestinal ischemia while exerting systemic hemodynamic effects (58). In a subsequent study by the same research group, cerebral tissue oxygen saturation <70% within the first 48 h of life was found to predict subjects at high risk of developing NEC, while intestinal fractional tissue oxygen extraction was elevated 2 days prior to the onset of NEC (59).

#### Conventional and Emerging Biomarkers

Considering the limitations of clinical and radiological findings in early diagnosis of NEC, research effort has focused on the discovery of biomarkers capable of predicting, early diagnosing, and discriminating NEC from non-NEC intestinal diseases. Currently, used biomarkers can be classified into three categories: (a) hematological indices, such as total white blood cell count, absolute neutrophil count, immature to total white blood cell ratio (I:T ratio), and platelet count; (b) acute phase reactants; and (c) immunological markers, including cytokines, chemokines, adhesion molecules, intracellular signal transduction molecules, and growth factors (60, 61).

Plasma, urine, and fecal levels of inflammatory mediators and molecules involved in immune response that have been evaluated as potential biomarkers for diagnosis and prediction of NEC include acute phase proteins (C-reactive protein, procalcitonin, serum amyloid-A [SAA], platelet activating factor, hepcidin), toll-like receptors, cytokines (IL-6, IL-8, TNF-α), and chemokines. These biomarkers are non-specific for NEC, and therefore they cannot differentiate NEC from other inflammatory diseases of the neonate. Their diagnostic accuracy varies depending on NEC severity and time-point during the course of the disease (62).

#### Most Promising Biomarkers for NEC

Among the biomarkers evaluated for prediction and diagnosis of NEC, fecal calprotectin (CP), intestine-fatty acid binding protein (I-FABP), claudins, and Trefoil factor 3 (TFF3) are the most promising for NEC diagnosis.

CP is a cytosolic protein, a member of the S-100 family, that is released by neutrophils and other inflammatory cells during inflammation. Increased fecal CP levels is an indicator of gastrointestinal inflammation. A meta-analysis of 13 studies including a total of 601 neonates exploring the value of fecal CP in NEC diagnosis found that fecal CP is markedly increased in infants with NEC. The reported sensitivity of fecal CP ranges from 76 to 100%, and the specificity ranges from 39 to 96% depending on the cut-off level. The authors concluded that despite the increased fecal CP levels found in NEC patients, its use as an early marker of NEC has not yet been determined (63). In line with this conclusion, a recent case-control study including 10 NEC preterm infants and 30 controls found wide inter- and intra-individual variations of fecal CP in preterm infants during the 1st weeks of life, which limits the utility of serial fecal CP measurements for prediction and early diagnosis of NEC (64).

I-FABP is expressed exclusively by enterocytes and is released in the blood following epithelial cell injury and excreted in the urine. An early study showed that increased I-FABP combined with levels of liver-FABP and TFF3 could differentiate infants with NEC from those with sepsis and the controls with a specificity of 95% or more and sensitivity of 50% as well as between medical and surgical NEC with a sensitivity of 83% and a specificity of 100% (65). A recent study showed that urine I-FABP in neonates with suspected NEC could discriminate neonates who subsequently developed NEC from those who developed sepsis and those served as controls (66). Moreover, elevation of postoperative I-FABP serum levels in infants subjected to cardiac surgery was associated with subsequent development of NEC (67). Two metanalyses showed that I-FABP levels in plasma and urine have a high specificity (91 and 73%, respectively), but moderate sensitivity (64 and 64%, respectively), which limits its value as a NEC biomarker (68, 69). However, a more recent review concluded that existing data overall suggests that the I-FABP is a promising biomarker for NEC with the potential to improve its diagnostic value when combined with other markers of intestinal damage (70).

Claudins are tight junction proteins that have been advocated as potential biomarkers for intestinal tight junction integrity. There are two published studies that examined the potential role of claudins in the early diagnosis of NEC. Both studies found decreased claudin−2 expression in the intestinal tissue from three preterm infants with NEC (71) and a spike of increased excretion of claudin 3 in the urine (72). However, existing data are insufficient to support a prognostic and diagnostic role of claudins in NEC.

TFF3 is another gut barrier protein expressed predominantly by the intestinal goblet cells. This factor is important for maintenance of mucosal barrier integrity, promotion of mucosal barrier repair and cell migration. TFF3 is released into the bloodstream following intestinal mucosal injury (73). Its involvement in gastrointestinal inflammation renders trefoil factor 3 non-specific to NEC. However, a combination of trefoil factor 3 with other biomarkers of intestinal injury and inflammation (e.g., I-FABP and either the liver-FABP or SAA) has been demonstrated to be an early predictor of NEC outcome (65, 66).

Excellent review articles on conventional and emerging biomarkers for NEC diagnosis that have been published are recommended for readers interested in more details (34, 60, 62, 74).

## Metabolomics and Proteomics Technologies

### The Role of Proteomics and Metabolomics in Understanding Disease Pathophysiology

From the five subfields of omics technologies that are integrated under the aspect of systems biology, proteomics and metabolomics have been shown to play an increasingly important role in screening, diagnosis, and prognosis, in advancing biomarker discovery while providing evidence of propelling the mechanistic investigation of the etiology of diseases (75).

The proteomic profile of an organism is a dynamic reflection of genes, environmental impact, and other factors, and as such the information we gain from proteins and peptides holds a great potential for biomarker discovery, especially for disease biomarkers, since proteins are most likely to be ubiquitously affected. However, proteomics studies are highly complicated due to the large complexity of the proteome (>1,000,000 proteins) and the difficulties in identifying low-abundant proteins and following post translational protein modifications.

Metabolomics have several advantages over the other omics approaches. In contrast to genomics, in which a genetic mutation may have little or no impact on the function of a protein and the final expression outcome in the organism, metabolomics detects the direct result of a biochemical response to a stimulus. Metabolites are the final downstream products of gene transcription, thus the impact on the metabolome is theoretically amplified relative to that of the transcriptome and the proteome. Moreover, metabolites are considered to represent the smallest domain (~100,000 metabolites), and as downstream products of gene transcription and protein translation, metabolites provide a closer image of the phenotype of the organism studied (76).

Proteomics and metabolomics strategies are thus applied to obtain a deeper understanding of biological phenomena based on the discovery of new knowledge via a hypothesis-free but necessarily hypothesis-generating approach. The methodology seeks to provide better understanding of normal physiological processes or of the onset and progression of disease or the (close to real time) response to therapeutic intervention.

### Prerequisites for Application of Omics Analysis

Typically, a proteomic or a metabolomic study requires multidisciplinary research and collaborative efforts by scientists from different fields of expertise including medicine, advanced analytical chemistry, biology, biochemistry, and biostatistics, while multiple steps have to be followed. The most critical step in these methodologies is the study design and sample collection. The generation of reliable data is based on the meticulous design of the experimentation parameters, taking into account the sample size in order to ensure statistical significance, the methods for sampling and sample handling, the selection of analytical technology, the data treatment strategy, and so forth (77). Strict criteria and standard operating procedures in sample collection and handling are required, especially in large or multi-center studies. The study population should be homogeneous, and, for cases involving humans, factors such as age, gender, ethnicity, body mass index, diet, lifestyle, drug therapy, diurnal variation, and the environment should be carefully considered and controlled.

Virtually any type of biological specimen can be assayed and provide a profile in a holistic approach. Blood, urine, or tissues are most explored; other sample types, such as saliva, feces, or cell cultures, are also collected to obtain information. It is expected that variations in the metabolome or proteome of biofluids and tissues will provide insight into the manifestation and progression of the disease (77).

In a proteomic experiment sample, treatment is a more composite procedure, as sample purification and protein digestion, plus affinity capture and fractionation, for example by the use of gel-based or chromatographic techniques, to reduce the complexity of the sample, are part of the typical workflow.

In metabolomics experiments, sample treatment is more direct and typically more straightforward and rapid. The basic concept is that less sample treatment, translates into a more comprehensive metabolic fingerprint (78, 79).

### Analytical Methods for Proteomics and Metabolomics

The major analytical platform for proteomics is liquid chromatography-mass spectrometry (LC-MS), whereas for metabolomics different techniques can be applied to address the different classes of small molecules and obtain a more complete image of the metabolome, including proton nuclear magnetic resonance spectroscopy (^1^H (NMRS), LC-MS and gas chromatography-mass spectrometry (GC-MS) (80).

Each of these techniques offer unique advantages in terms of detection sensitivity, resolution, accuracy, reproducibility, dynamic range, and throughput; depending on the target molecules and the desired task, the appropriate platform should be selected. In reality, in order to analyze the entire proteome or metabolome a combination of several techniques is needed.

Whichever analytical method implemented, special attention should be paid to quality assurance (QA) and quality control (QC) in the analytical processes, especially for large-scale studies (81).

### Advances in Omics Technology and Future Perspectives

In the last decades, new advances in analytical technologies have boosted the omics fields, providing not only increased instrumental performances and capabilities but also more powerful data analysis software—an essential tool for looking deep into the complex data.

The evolution (or for some researchers the revolution) that has occurred in the omics fields would not be possible without the evolution in computing ability. Bioinformatics develops and exploits advanced statistical analysis tools such as various sorts of multivariate statistics, machine learning, and artificial intelligence. The final scope is to reveal and validate molecules or patterns that are characteristic of the studied physiology and subsequently to validate them. Integrating such biomarkers in a life science context is equally important: biomarkers should subsequently be related to physiology and linked to one or more biochemical pathways of the studied organism/cell. In this aspect, computational analysis of biochemical pathways and networks may also employ large scale databases, such as proteome data banks, or metabolomics databases.

As a future aspect, these two omics approaches may enable us to develop new tools of predictive, preventive, and personalized value in clinical and medical sciences. On the other hand, omics share several limitations such as the inherent difficulty in comparing and merging of data obtained in different settings. Currently, harmonization of methods and protocols is still inadequately applied among proteomics and metabolomics researchers. Significant efforts are necessary to reach appropriate standardization, particularly in untargeted LC-MS methods. This should expand to reporting analytical data and meta-data (79). Finally, the development and advancement of public databases can pave the way for more sophisticated developments in the future.

## NEC Biomarker Discovery Using the Metabolomics and Proteomics Approach

During the past decade, emerging molecular techniques have been utilized in order to further comprehend the pathophysiology of NEC and discover surrogate biomarkers. Currently, proteins, metabolites, and other molecules that have surfaced using metabolomics and proteomics are being evaluated as biomarkers of NEC.

### Specimens Used for Metabolomics and Proteomics

Metabolomics and proteomics can be applied to a wide variety of biological fluids and tissues, including serum, plasma, urine, cerebrospinal fluid, amniotic fluid, saliva, bronchoalveolar fluid, feces, gastric fluid, and surgically resected intestinal tissue. Important factors determining the biological specimen of choice include the purpose of analysis, that is, disease state or target-organ, the properties of the metabolite under investigation, and the subject's characteristics. Serum and plasma are the specimens most often recruited for omics analysis. However, the high protein content of serum and plasma, often limit the study of serum biomarkers. In neonates, especially the VLBWI who are the most frequently affected by NEC, there are additional concerns in obtaining blood samples; the pain and stress caused by blood sampling, especially when repeated multiple times, may have short- and long - term consequences, while there are certain restrictions regarding the amount of blood allowed to be drawn. Therefore, the use of non-invasive samples including urine, stool, gastric aspirates, saliva, and buccal swabs is preferred. Urine is an advantageous fluid for metabolomics and proteomic analysis for three main reasons; firstly, many intermediate products of metabolism reflecting the function of many organs and systems are excreted in urine; secondly, urine is a plasma filtrate, thereby simpler than plasma, containing only small molecules; and finally, urine samples can be obtained easily from neonates using a cotton ball (82–84). However, concerns have been raised regarding the potential contamination of urine samples by cotton-derived material (85, 86). Stool is also a non-invasive material for metabolome/proteome analysis. However, in preterm neonates, bowel movements may be delayed for several days, making omics-related early prediction of NEC problematic (87, 88). The amniotic fluid, which reflects the intrauterine milieu and fetal metabolic profile, may rise into a biologic fluid with important predictive properties in the context of the association between NEC and maternal risk factors, mainly chorioamnionitis (89). The omics analysis of amniotic fluid could reveal several metabolites, proteins, and genes associated with preterm delivery, intrauterine growth restriction, intrauterine inflammation, infection, and other pregnancy-associated morbidities (90, 91). Previous studies showed that the metabolomic profile of amniotic fluid could predict neonates at high risk of developing morbidities associated with intrauterine environment, such as sepsis, perinatal asphyxia, and BPD, while, theoretically, amniotic fluid metabolomics could provide insight regarding NEC development and adverse neurodevelopmental outcome (92–95). The value of amniotic fluid as a source for biomarker discovery is limited by the invasive way of acquisition via amniocentesis, as noted in most published relevant studies. However, amniotic fluid can be obtained during delivery or cesarean section, rendering a non-invasive specimen potentially useful for early prediction of infants at high risk of developing NEC. Gastric fluid at birth is a mixture of amniotic fluid and gastric secretions reflecting both the intrauterine environment and gastric function of the fetus (96). So far, gastric fluid has not been used as a predictive tool for neonatal morbidities, despite the fact that it can be obtained non-invasively through an orogastric tube, which is often used in delivery practice. In addition, buccal swabs have been used for proteome analysis in preterm infants with good discriminating capacity between NEC cases and controls (88). Metabolome/proteome analysis in bronchoalveolar fluid samples obtained during routine endotracheal suction from intubated neonates could offer important information on two major neonatal problems: respiratory distress syndrome and the risk of developing BPD.

### Metabolomics and Proteomics Studies in Necrotizing Enterocolitis

Plasma/serum and urine metabolomics and proteomics have been studied in healthy neonates as well as in various neonatal conditions including, but not limited to, perinatal asphyxia, patent ductus arteriosus, inborn errors of metabolism, sepsis, and NEC (97–100). Current data regarding the detection of surrogate biomarkers for prediction, diagnosis, and prognosis of NEC derived from experimental and clinical studies using metabolomics and proteomics approach is limited (Table 2).

**Table 2:** Clinical studies on metabolomics and proteomics in infants with necrotizing enterocolitis.

**References**	**Settings/study design**	**Subjects**	**Methods**	**Specimens - timing**	**NEC diagnosis criteria**	**Main findings**	**Major conclusions**
Ng et al. 2010 (101)	Single center, case - control; and validation, cohort study	PTI with BW < 1,500 g; Part 1 (case-control): 77 sepsis/NEC and 77 controls. Part 2 (validation cohort): 60 sepsis/NEC and 40 controls.	Proteomics using targeted MS.	Serum at clinical presentation (10 cases & 10 controls longitudinally)	Symptoms, full sepsis screening, antimicrobial coverage.	Pro-apoC2 and SAA were the most promising biomarkers. The ApoSAA score was effective in identifying sepsis/NEC cases and stratification of infants into different risk categories	The ApoSAA score can potentially be useful for early and accurate diagnosis of sepsis/NEC
Morrow et al. 2013 (87)	Single center, case-control	11 infants who developed NEC and 21 matched controls <29 wks	Fecal microbiome using 16S rRNA. Urine metabolomics using ^1^H NMRS.	Stool (DoL 4-9 & 10-16) and urine (DoL 4-9) collected prior to disease	Bell's stage II-III	NEC cases were dominated by firmicutis and proteobacteria. Metabolome profile did not differ between NEC cases and controls.	No single or cluster of urine metabolites discriminated between NEC and non-NEC cases
Jiang et al. 2013 (102)	*Ex vivo* study NEC- tissue vs. adjacent healthy tissue proteome	10 neonates 24–34 wks GA	Proteomics using 2D-DIGE, MS, Western blot, and immunohistochemistry	Small intestinal and colon tissues	Bell II - III operated	Expression of 30 and 23 proteins of small intestine and colon, respectively, differed significantly between NEC- affected vs. vital sections	The proteome changes in NEC-affected tissues provide a basis for targeted proteomics to detect plasma biomarkers.
Torrazza et al. 2013 (88)	Multicenter, case-control	VLBWI (<32 weeks & <1,250 g). *Phase* 1: 3 NEC & 3 controls; *Phase* 2, validation: 10 NEC & 10 controls	Proteomics profiling by 2D-DIGE; LC-MS/MS proteomics platform; validation using Western blot	Buccal swab samples, at 1, 2 and 3 weeks prior to the development of NEC	x-ray signs or intraoperative confirmation	NEC cases had 21 altered proteins (8 increased and 13 decreased, all *p* < 0.05) compared to controls. 3 biomarkers with high fold difference between NEC and controls (IL-1RA, peroxiredoxin-1, and α1 -antitrypsin).	IL-1r antagonist is worthy of further studies to determine its utility in helping predict NEC.
Sylvester et al. 2014 (82)	Multi-center, cohort	119 premature infants (85 NEC, 17 sepsis, 17 control)	Proteome profile using non-targeted LC-MS (45 NEC, 12 sepsis, 2 controls); biomarker validation using ELISA (40 NEC, 5 sepsis, 15 controls)	Urine obtained at suspicion of NEC or sepsis.	Bell's criteria (pneumatosis intestinalis)	A panel of 7 biomarkers was identified and validated. ROC AUC 98% for NEC vs. sepsis, and 98.4% for medical NEC vs. surgical NEC	The 7 - protein panel provides an accurate diagnosis and prognosis for infants with suspected NEC.
Sylvester et al. 2014 (103)	Multi-center, cohort	550 preterm infants with suspected NEC, including 65 with urine metabolomics	Algorithm of 27 clinical/radiological/ hematological parameter and urine peptide profile	Urine obtained at suspicion of NEC	Clinical findings and pneumatosis intestinalis	An algorithm of 27 clinical parameters combined with three urine peptides associated with intravascular coagulation could accurately predict all cases with surgical NEC	Combining clinical parameters with biomarkers improves prediction of progressive NEC
De Magistris et al. 2015 (104)	Preliminary, longitudinal, case-control	6 NEC cases and 12 controls	Urinary metabolomic profile using ^1^H NMRS	Serial urine samples from birth to 40-60 DoL	-	Increased urinary gluconic acid in NEC cases.	The low numbers did not allow conclusion to be drawn.
Stewart et al. 2015 (105)	Single center, case -control.	10 NEC/LOS cases, 9 controls.	Proteomics using Micro BCA protein assay kit. Metabolomics using a multistep LC-MS/MS.	Serial serum samples at 14 ± 7 d and 1 ± 5 d prior to diagnosis and 14 ± 4 d after diagnosis.	Confirmed NEC and/or LOS.	Several proteins were associated with disease status. No distinct profile that could predict patients NEC or LOS.	It is unlikely a single biomarker could discriminate NEC and/or LOS from healthy infants.
Stewart et al. 2016 (106)	Single center, case-control.	7 PTI who developed NEC and 28 controls.	Microbiomics by 16S rRNA. Metabolomics by UPLC-MS/MS on a subset of 6 NEC and 10 controls.	Stool, daily sampling.	Clinical & x-rays findings.	Defined 6 clusters of gut microbiome. Clusters 1–5 were associated with NEC development. Metabolites involved in linoleate metabolism were associated with NEC.	The metabolome and microbiome profiles were highly concordant, while metabolites associated with NEC were significantly reduced in the healthy preterm gut community types.
Wilcock et al. 2016 (107)	Single center, case-control	PTI <30 wks *n* = 12 (7 with NEC) vs. term controls *n* = 8	Metabolomics using GC-MS	Serum; sample A: 1st week of life; sample B: at full enteral feeding	Clinical and x-rays findings	Both A and B sample metabolome differed between preterm and term groups. The B sample metabolome differed significantly between NEC- & non-NEC-cases	Metabolomics differed in PTI at risk of NEC at the age of full enteral feeds. The study was not powered to determine a NEC-biomarker.
Rusconi et al. 2018 (108)	Single center, prospective cohort.	PTI, BW < 1,500 g: 24 with Bell's Stage ≥ II NEC, 5 Bell's Stage I NEC, and 67 controls.	Untargeted and targeted metabolomics using UPLC-MS/MS.	Stool collected 1–5 d before NEC development.	Bell's stage II or III NEC.	Ceramides were decreased and sphingomyelins increased in NEC cases Bell's stage II & III.	Broad application of sphingolipids predictive biomarkers needs attention.
Wandro et al. 2018 (109)	Single center, prospective cohort.	32 VLBWI (3 NEC & 8 sepsis & 21 controls) over the first 6 weeks of life.	Microbiome using 16S rRNA. Metabolome using untargeted GC-MS.	Stool collected over the first 6 weeks of life.	-	Fecal metabolomes were associated weakly with bacterial composition but not NEC, LOS, or health outcomes.	Fecal metabolome is not associated with NEC or other health outcome.
Chatziioannou et al. 2018 (110)	Single center, case -control.	PTI, 25 NEC, 18 LOS, equal number of controls.	Targeted LC-MS/MS, 47 protein markers.	Serum, d 0, 3, and 10 after the onset of LOS or NEC.	Bell's criteria for NEC; clinical & hematology/ serology/ culture criteria of probable or proven sepsis.	Two panels of three proteins each displayed a high discriminating ability; a panel of CRP, CETP, and apoA-IV for LOS vs. control (ROC AUC = 0.98) and a panel of apoA-VI, ApoC1, and LCAT for NEC vs. LOS at D0 (ROC AUC = 0.999).	Two panels of three proteins each exhibit an excellent diagnostic performance at the LOS-NEC and LOS-control discrimination.
Thomaidou et al. 2019 (111)	Single center, case - control.	PTI, 15 NEC (5 Bell's stage I and 10 stage II/III) & 15 GA-matched controls	Non-targeted ^1^H NMRS and targeted LC-MS monitoring 108 metabolites.	Urine obtained at diagnosis of NEC	Bell's staging I - III	25 discriminant metabolites were identified belonging to amino acids, organic acid, sugars, and other.	Certain urine metabolites could discriminate between NEC and non-NEC cases thereby being candidate biomarkers for NEC.
Sinclair et al. 2020 (112)	Prospective, multicenter, longitudinal, cohort study.	73 infants with NEC and 814 controls.	Tandem MS; 72 amino acid and acylcarnitine.	Dried blood spot; serial on 1, 2–7, 8–28, and 29–42 DoL.	Clinical & x-rays criteria	Several AA and ACs differed significantly between NEC infants and controls at each time point. The number of deviating metabolites increased progressively as NEC onset approached.	The early appearance of metabolic deviation in NEC cases highlights the predictive potential of the metabolites assessed in this study.

*16S rRNA, 16S rRNA gene sequencing; 2D-DIGE, 2-Dimentional Difference Gel Electrophoresis; AA, amino acid; AC, acylcarnitine; d, day(s); DoL, day of life; BW, birth weight, GA, gestational age; GC-MS, Gas Chromatography - Mass Spectrometry; IL-1RA, Interleukin-1 Receptor Antagonist; LC-MS, Liquid Chromatography - Mass Spectrometry; LOS, late onset sepsis; MS, Mass Spectrometry; NEC, necrotizing enterocolitis; ^1^H NMRS, proton Nuclear Magnetic Resonance Spectroscopy; Pro-apoC2, proapolipoprotein CII; PTI, preterm infants; ROC AUC, receiver operating characteristic area under the curve; SAA, serum amyloid A; UPLC-MS, Ultra-Performance Liquid Chromatography - Mass Spectrometry; VLBWI, very low birth weight infants*.

#### Preclinical Studies

Several experimental studies have investigated the plasma/serum and intestinal epithelial cell metabolome and proteome in experimental models of NEC to decipher the pathogenesis of NEC and detect candidate biomarkers.

Experimental studies in mice by Romick-Rosendale et al. (113) explored the role of antibiotics in NEC development using urine and fecal metabolomics. Clusters of fecal and urine metabolites were discovered that could discriminate subjects with antibiotic-induced intestinal damage from controls and those with repaired gut damage after antibiotic discontinuation. The authors suggested the urinary trans-4-hydroxy-L-proline, reflecting the damage in the connective tissue of ileal villi, as a surrogate biomarker for antibiotic-induced intestinal injury (113).

A Hong Kong/China/Denmark research team conducted a series of five studies in a preterm pig model of formula feeding-induced NEC exploring intestinal microbiome as well as plasma, urine, and intestinal proteome and metabolome profiles in NEC. The main results of these studies can be summarized as follows: (a) NEC development was associated with changes in expression of 19 proteins, which are involved in several metabolic and inflammatory processes (114); (b) the second study demonstrated that the serial changes in the expression of 25 intestinal proteins, which are involved in important cellular functions and metabolic pathways, were associated with early progression of NEC (115); (c) a subsequent study showed that 14 intestinal proteins were differentially expressed between germ-free and conventionally reared pigs, indicating that the initial bacterial colonization may induce stress response rendering the immature intestine sensitive to the pro-inflammatory effects of enteral feeding (116); and (d) finally, two experimental studies explored the effect of early, short-term antibiotic administration on NEC development using the proteomic approach. Intestinal histology revealed a lower incidence of NEC while the intestinal proteomic profile was compatible with attenuated inflammatory response in the antibiotic-treated pigs (42). Moreover, plasma and urine metabolomics showed significant alterations in several metabolites, which were attributed to reduced gut inflammation, increased intestinal permeability, and altered host–microbiome interaction in the antibiotic treated animal models of NEC (41).

Another research team from Denmark assessed the intestinal proteome profile in the context of exploring the potentially protective effect against NEC development of certain factors in human milk, namely lactoferrin and transforming growth factor 2 (TGF2). An *in vitro* study demonstrated that TGF-β2 induced the differential expression of 13 proteins, most of them associated with stress responses as well as TGF-β2 and Toll-like receptor 4 signaling pathways, supporting a regulatory effect of TGF-β2 on intestinal epithelial cell responses against microbial invasion (117). In a following study, the enterocyte proteome profile was used as a marker of intestinal integrity and function in pigs fed formula supplemented with bovine lactoferrin. A dose-dependent effect of lactoferrin on intestinal proteome was found, compatible with a potentially detrimental effect of high doses of lactoferrin on gut function (118).

More recently, Call et al. (119) performed microbiome and metabolome analysis in the cecum and plasma, respectively, of a piglet model of NEC exploring the effect of lactose- and corn syrup-based formula. They found that lactose-based formula-fed piglets was associated with a lower prevalence of NEC compared to corn syrup-based- or mixed formula-fed piglets. Plasma from NEC-piglets was enriched with products of purine and aromatic amino acid metabolism and bile acids despite the modest changes observed in the gut microbiome profile (119).

Overall, existing experimental data suggests that certain clusters of metabolites involved in inflammatory response, cell apoptosis, oxidative stress, signal transduction, and energy metabolism, differ significantly between NEC models and controls. Although results of experimental studies have shed further light on the pathogenesis of NEC, the pattern of the identified proteins and other metabolites is inconsistent to the point that it does not allow identification of certain proteins or clusters of them that could be further evaluated as surrogate biomarkers of NEC in the clinical setting.

#### Clinical Studies on Metabolomics in Necrotizing Enterocolitis

Morrow et al. conducted a prospective, case-control study in two secondary level neonatal units, with the aim of identifying early microbial and metabolic biomarkers associated with NEC development. Eleven infants with GA < 26 weeks and BW < 1,200 g who developed NEC (Bell's stage II and III) and 21 matched controls were included. Metabolomics and metagenomics were assessed in the first 2 weeks of life in 60 urine samples and 58 stool samples, respectively, obtained from NEC neonates, non-NEC deaths, and controls, utilizing ^1^H NMRS analysis and 16S rRNA gene sequencing, respectively. It was found that firmicutes or proteobacteria colonization preceded the development of NEC while propionibacterium was found only in control samples. Urine metabolomics did not reveal any significant difference in metabolome profile between NEC cases and controls. Further analysis showed that changes in individual urine amino acids were associated with the microbiome preceding the development of NEC. Specifically, urine alanine was significantly higher in NEC preceded by firmicutes (*p* < 0.001) while histidine was significantly lower in NEC preceded by proteobacteria dysbiosis (*p* = 0.013). However, evaluation of urine alanine and histidine as surrogate markers for NEC showed that none of these amino acids alone was significantly associated with overall NEC risk. On the other hand, a ratio of alanine to histidine over four produced a predictive value of 78%, with a sensitivity of 82% and a specificity of 75% (87). In summary, this early study highlights the association between the pattern of bacteria colonization and certain urine amino acids and provides evidence supporting the involvement of early dysbiosis in NEC development. In addition, results indicate the microbial profile and the ratio of urine alanine to histidine as potential biomarkers for prediction of NEC.

De Magistris et al. performed a preliminary study to investigate temporal changes in urine metabolome profile using ^1^H NMRS. The study population consisted of six infants who developed NEC and 12 age-matched controls. Urine samples were collected from birth through the 40–60th day of life. Urine metabolome profiles in NEC cases were affected by the day of disease onset. A constant pattern of metabolome serial changes was observed in all recruited infants, except for the cases of NEC occurring after the 40th day of life. In NEC cases, increased urinary gluconic acid levels were found, which can be attributed either to the gluconic calcium administration or just be a byproduct of glucose oxidation or microbiome metabolism (104). In summary, results of this study indicate that the age at NEC onset and the day of the disease should be taken into account when evaluating urine metabolites as potential biomarkers for NEC diagnosis. A limitation of the study is the small study sample that warrants cautious interpretation of the findings.

Stewart et al. conducted a case-control study aiming to explore the serial changes in serum proteome and metabolome in preterm infants with NEC or late onset sepsis (LOS) in comparison with matched controls. The study population consisted of 19 preterm infants with GA of 23–29 weeks receiving intensive care, nine with NEC, and 10 controls matched for GA and age at sampling. Serum samples were collected at three time-points: 14 ± 7 days and 1 ± 5 days prior to disease diagnosis and 14 ± 4 days after disease diagnosis. Serum metabolomic profile was analyzed utilizing LC-MS and proteins using the Micro BCA protein assay kit (Pierce). Overall, 447 unique proteins and 24,153 metabolites were detected. Eight proteins were significantly associated with NEC and four proteins with LOS, while no protein was exclusively found in NEC patients. Among these proteins, C-reactive protein, Ig a-2 chain C region, and macrophage migration inhibitory protein were increased in a higher number of NEC patients (105). To summarize, the study did not identify any distinct metabolome profile or individual proteins and metabolites which could predict the NEC or LOS occurrence. Of interest is the finding that the proteins associated with NEC differed from those associated with LOS, suggesting that proteomics might differentiate between NEC and LOS without NEC.

Stewart et al. in a case-control study of seven neonates who developed NEC and 28 matched controls performed temporal bacterial and metabolomic profile of the gut microbiome in daily obtained stool samples. NEC was defined according to clinical and radiology data. Microbiome profiling of stool defined six distinct clusters (preterm gut community types): clusters 1–5 were present in NEC infants, while cluster 6, demonstrating a high diversity with bifidobacterial dominance, was present only in the control samples. Moreover, metabolites associated with NEC were increased in clusters 1–5 and decreased in cluster 6. Metabolomics revealed that certain metabolites involved in C21-steroid hormone biosynthesis, linoleic acid metabolism, and arachidonic acid pathways, were associated with NEC development (106). In summary, this study supported a protective effect of gut microbiome diversity with a high abundance of bifidobacteria on NEC development and revealed a number of metabolites associated with NEC that could be further evaluated as potential biomarkers for NEC prediction. Interestingly, unlike more recent studies, results of this study did not reveal any distinct gut microbial signature in NEC cases or a significant effect of GA on gut microbiome (33, 87).

Wilcock et al. performed an observational metabolomic study to explore the value of metabolomics in discovering biomarkers capable of reliably detecting preterm infants at high risk for developing NEC. The study population consisted of 12 neonates with a GA of < 30 weeks, including seven infants who subsequently developed NEC, and eight term controls. Serum metabolomics were assessed using GC-MS at two time points; within the 1st week of life and at the time of full enteral feeds. NEC -associated metabolites were used to construct a network model including all known interactions related to the genomic function using the Ingenuity Knowledge Base (IKB). Metabolomics at both time points differed significantly between term and preterm infants. Moreover, metabolomics at the time of full enteral feeds, but not within the 1st week of life, differed significantly between NEC and non-NEC preterm neonates, but without holding sufficient discriminating ability (107). In summary, metabolomics in this study did not reveal any certain metabolites that could be used as biomarkers for NEC diagnosis, probably due to the small number of participants. The differences in metabolomic profile between the total preterm group and the term neonates could be attributed to either the different mode of nutrition (parenteral and enteral in preterm and term infants, respectively), or NEC manifested in 7/12 preterm infants. Moreover, the fact that no metabolite had sufficient discriminating ability despite the considerable metabolic differences observed between NEC patients and control infants (up to 8-fold change) at the time of full enteral feedings underlines the need for large multicenter studies.

Wandro et al. in a retrospective cohort study evaluated longitudinally gut microbiota changes and metabolome in 32 VLBWI (BW < 1,500 g) aiming at better understanding of the assembly evolution of gut microbiota in preterm infants. For this purpose, fecal samples were collected from each infant over the first 6 weeks of life. Fecal bacterial composition was assessed using the 16S rRNA gene sequencing while metabolomics analysis was performed using untargeted GC-MS. During the study period, 3/32 infants developed NEC, 8/32 developed LOS. Longitudinal assessment revealed significant changes in the microbial composition over time. Metabolites detected in fecal samples included metabolic products of amino acids, lipids, nucleotides, and vitamins, fermentation products, bile acids, fatty acids, organic acids, sterols, sugars, and other substances (109). In summary, this study showed that fecal metabolomes are unique for each individual and change over time, while they are not associated with NEC or other health outcomes. An important limitation is the extremely low number of NEC-cases.

Rusconi et al. prospectively studied the fecal metabolomic profile in a cohort of neonates with BW ≤ 1,500 g utilizing untargeted and targeted metabolomics. Among the participants, 24 had Bell's stage II -III NEC, 5 had Bell's stage I NEC, and 67 were controls matched for GA, BW, and the day-of-life. Samples collected 1–5 days prior to NEC onset were analyzed in nine NEC cases (nine samples) and 19 controls (32 samples). Untargeted metabolomics analysis identified more than 700 metabolites, consisting mainly of lipids, carbohydrates, amino acids, and xenobiotics. The targeted metabolomics analysis revealed that sphingomyelins were significantly higher and ceramides significantly lower in NEC cases with Bells' stage II-III, but not in stage I NEC. Conclusion was that, despite the significant differences in sphingolipid metabolism observed in pre-NEC stools compared to controls, broad application of sphingolipids as predictive biomarkers needs attention (108). In summary, changes in sphingolipids and ceramides were observed in Bell's stages II and III of NEC patients, an observation that needed further evaluation as surrogate biomarkers of NEC. The similar levels between Bell's stage I and controls, may support the view that the Bells' stage I represents a pre-NEC condition.

Thomaidou et al. utilized untargeted ^1^H NMRS and targeted LCMS to detect urine metabolites that could serve as potential biomarkers for NEC diagnosis. In this single center, prospective, case-control study, 30 neonates with GA 29–36 weeks, 15 with NEC and 15 GA-matched controls, were included. Of the NEC neonates, five had Bell's stage I and 10 Bell's stage II/III. Urine metabolomics were assessed at suspicion of NEC/sepsis. ^1^H NMRS and LCMS analyses revealed a total of 25 metabolites, including amino acids, organic acids, sugars, and vitamins, that differed significantly between NEC-cases and controls. A total of 14 metabolites had an excellent diagnostic performance in detecting neonates developing NEC, with a statistically significant area under the ROC curve (AUC). Among them, a set of three metabolites (tyrosine, arginine, riboflavin) displayed a significant diagnostic value (111). In summary, the results of the study suggest further evaluation of urine tyrosine, arginine, and riboflavin as surrogate biomarkers for NEC diagnosis. Unlike the study by Rusconi et al. (108), exclusion of Bells' stage I NEC cases did not change the results indicating that metabolic alterations observed in this study are detectable during suspected NEC and could be useful as early diagnostic biomarkers.

Sinclair et al. in a prospective, cohort study, based on a multicenter neonatal intensive care database collected by the Pediatrix-Obstetrix Center for Research, Education, and Quality explored the serial changes of amino acids and acylcarnitines during the first 6 weeks of life in relation to the development of NEC. The study population included 995 preterm infants (<32 weeks gestation) having four serial dried blood spots samples collected in the first 6 weeks of life. Of them, 73 preterm infants developed NEC while the 814 comprised the control group. A panel of amino acids and acylcarnitines was analyzed using tandem MS. It was found that early levels and serial changes of a number of metabolites were associated with the development of NEC (112). In summary, neonates who are about to develop NEC manifest early changes in blood amino acids and acylcarnitines that progressively deteriorate until the onset of NEC. These findings highlight the predictive potential of amino acid and acylcarnitine assessment. An important advantage of this method is the sparing of neonatal blood by using dried blood spots. The effect of nutritional intake on the metabolic changes observed in this study remains to be further elucidated.

#### Clinical Studies on Proteomics in Necrotizing Enterocolitis

Proteomics focus on the quantification of proteins and peptides, protein to protein interactions and post-translational modifications. Proteomics have been used in various clinical conditions including bowel disease and sepsis for discovering biomarkers that are candidate for further clinical validation. So far, there are six published clinical studies on the use of proteomics to elucidate NEC pathophysiology and potentially discover candidate biomarkers for NEC.

Ng et al. explored the potential of proteomics to detect surrogate biomarkers for neonatal sepsis and/or NEC. The study was conducted in two parts: a case-control study including 37 neonates with sepsis/NEC and 37 controls and a validation cohort study of 104 additional episodes of suspected sepsis/NEC. Plasma proteomic profiling was assessed at clinical presentation using MS-based technology. The case-control part of the study detected 65 proteomics peaks that differed significantly between NEC cases and controls. Among them, the pro-apolipoprotein CII (Pro-apoC2), a des-arginine variant of serum amyloid A (SAA), transthyretin, and α-1B-glycoprotein had a high discriminating ability, with the AUC ranging from 0.69 to 0.96. The ApoSAA score calculated from plasma apoC2 and SAA concentrations could effectively discriminate sepsis/NEC cases from controls, with a specificity ranging from 55 to 95% and a sensitivity from 90 to 100%, depending on the cutoff value. Implementation of the ApoSAA score in clinical practice enabled either reduction of antibiotic administration or early discontinuation in non-septic infants without the risk of withholding treatment in septic infants (101). In summary, four proteins were identified as the most promising biomarkers for NEC while the ApoSAA score was found to be a practical for clinical use as a tool allowing early and accurate diagnosis of sepsis/NEC. The fact that the study did not make a distinction between sepsis and NEC cases along with the analysis of proteome at the onset of manifestations limits the utility of the ApoSAA ratio as an early biomarker of NEC in the absence of sepsis.

Jiang et al. utilized MS to assess the proteomics profile of small intestinal and colon tissues that were removed from 10 preterm infants (GA 24–34 weeks) with surgical NEC of Bell's stages IIA to IIIB. The expression of 30 proteins in intestine and 23 proteins in colon was significantly different between the NEC affected and the near-normal sections. Five proteins' expression, namely the histamine receptor subunit peptide, actins, globins, immunoglobulin, and α1-antitrypsin, differed between NEC and the respective normal tissue in both the small intestine and the colon (102). In summary, this study revealed five proteins that were differentially expressed between the NEC-affected intestinal/colon tissues and the neighboring normal tissues. Should plasma levels of the affected proteins reflect their perturbations in NEC tissues, they could be evaluated as biomarkers for diagnosis of advanced NEC. The limitation of the study was the fact that the control tissues were mildly affected by NEC.

Torrazza et al. in a multi-center case-control study of preterm infants with GA <32 weeks and BW <1,250 g integrated bioinformatics and proteomic approach to investigate the utility of buccal swab proteomics in discovering biomarkers for NEC prediction and diagnosis. To this aim, buccal swab samples were obtained from 13 infants at 1, 2, and 3 weeks prior to the development of NEC and from 13 matched controls. The study was conducted in two phases: a detection phase using LC-MS/MS analysis and a validation phase using Western blot analysis. The detection phase revealed a total of 21 proteins (8 increased and 13 decreased) that differed significantly between NEC-cases and controls. Further analysis of data using the PANTHER bioinformatics software and the Pathway Studio software showed that the altered proteins are involved in metabolic, inflammatory, and oxidative stress networks associated with NEC pathogenesis. Three of the proteins, namely the IL-1 receptor antagonist, peroxiredoxin-1, and α1 -antitrypsin, that differed significantly between NEC and non-NEC infants were further validated. The validation study showed that only the interleukin-1 receptor antagonist trended lower (*p* = 0.08) within 2 to 3 weeks before the onset of NEC compared to controls (88). In summary, the proteomics analysis of buccal swabs identified three proteins that were significantly associated with NEC, while the validation study revealed only the interleukin-1 receptor antagonist as a candidate marker for further evaluation. An important advantage of this study is the use of a non-invasive and easily obtainable biological specimens.

Sylvester et al. conducted a multi-center, cohort study using an exploratory proteomics approach in order to define a panel of urine proteins that could be used for early diagnosis and prognosis of NEC. The study consisted of two phases: the discovery and the validation phase. In the discovery phase, untargeted urine proteomics analysis was performed in a cohort of 45 NEC, 12 septic, and two control infants using non-targeted LC-MS. In the validation phase, urine samples of 40 NEC, five septic, and 15 healthy control infants were analyzed using enzyme-linked immunosorbent assay (ELISA) to quantify the urine protein biomarkers identified in the discovery phase. The discovery phase identified a panel of seven urine biomarkers, namely, alpha-2-macroglobulin - like protein 1, cluster of differentiation protein 14, cystatin 3, fibrinogen alpha chain, pigment epithelium-derived factor, retinol binding protein 4, and vasolin, which were consistently altered in NEC infants. On validation phase, the panel of seven biomarkers displayed a high discriminating ability between NEC and sepsis (AUC 98.2%) as well as between medical and surgical NEC (AUC 98.4%) (82). In summary, detection and validation parts of this study revealed a panel of seven proteins, a finding that is promising as a potential diagnostic and prognostic tool for infants with suspected NEC.

In a subsequent, multicenter study, Sylvester et al. assessed the diagnostic value of an algorithm consisting of 27 “clinical” parameters, which in fact included clinical as well as conventional radiological and hematological parameters, combined with urine peptides detected and validated using LC/MS. The study population included 550 neonates with suspected NEC, among them 65 with urine proteomics. The detection and validation studies suggested a panel of three fibrinogen alpha-chain-derived peptides reflecting intravascular coagulation which were able to accurately discriminate between medical and surgical NEC. Integrating both the algorithm and the panel of peptides enabled the detection of all infants with surgical NEC (103). In summary, this study showed that an algorithm consisting of 27 NEC-associated clinical, radiological, and conventional hematological parameters combined with a panel of three peptides associated with intravascular coagulation could accurately predict the progression to surgical NEC.

In a prospective case-control study, Chatziioannou et al. evaluated the utility of plasma proteome profile in differentiating between sepsis and NEC in preterm infants. To this aim, proteome was assessed in 25 infants with NEC (five with suspected, 19 with confirmed, and one with advanced NEC), 18 with LOS (seven possible and 11 confirmed LOS), and an equal number of matched controls. Serial plasma samples were obtained at disease clinical suspicion and on days three and ten thereafter. Assessment was performed using a novel selected reaction monitoring (SRM), targeted LC-MS/MS method. Statistical analyses revealed two panels of three proteins each having high discriminating ability; a panel of C-reactive protein, cholesteryl ester transfer protein (CETP), and apolipoprotein A-VI (apoA-IV) could differentiate between LOS at the onset of sepsis and controls (AUC = 0.98), while a panel of apolipoprotein A-VI, apolipoprotein C1, and lecithin-cholesterol acyltransferase discriminated NEC from LOS at the time of sepsis suspicion with excellent accuracy (AUC = 0.999 with 95% CI (0.987–1.00) (110). In summary, this study suggests two panels of three proteins each for further evaluation as surrogate biomarkers for discriminating sepsis from NEC and controls, respectively.

## Discussion

Prediction and early diagnosis of NEC is of utmost importance when trying to reduce the burden of this catastrophic disease. Properties of an ideal biomarker include molecular stability, rapid increase, preferably prior to clinical manifestations, and rapid decline following disease improvement. Additionally, an ideal biomarker for NEC should be non-invasive, specific for intestinal inflammation, with the ability to discriminate between NEC and other, non-NEC diseases. No single biomarker or cluster of biomarkers meets all the above criteria (51, 120).

So far, there are only a few published clinical studies dealing with the discovery of biomarkers for NEC prediction and diagnosis using the metabolomic and proteomic approach. Specifically, review of the literature revealed 15 clinical studies reporting on metabolomics and proteomics potential in discovering NEC biomarkers published within the last 11 years. Among them, five studies dealt with metabolomics (104, 107, 109, 111, 112), six with proteomics (82, 88, 101–103, 110), while four studies integrated metabolomics with either proteomics (one study) (105) or metagenolics (three studies) (87, 106, 109). A total of 349 infants with either suspected or definite NEC, 147 infants with sepsis/NEC, and 1,164 matched controls were included (Table 2). The most commonly used specimens for omics analysis were urine in five studies (87, 103, 104, 111, 112), stool in four studies (103, 105, 108, 109), and plasma/serum in four studies (101, 105, 107, 110), while surgically excised intestinal/colon tissues, dry blood spots, and buccal swabs each was used in one study, respectively (88, 102, 112). These studies detected several molecules that differed significantly between NEC cases and controls. The diagnostic value including positive predictive value, sensitivity, specificity, and/or AUC has been reported by only five clinical metabolomics and proteomics studies (82, 87, 101, 103, 110).

Nevertheless, there is a high inconsistency among studies with regards to the isolated biomarkers or panels that could be candidates for further evaluation as NEC biomarkers.

### Strengths and Limitations of Metabolomics and Proteomics in Detecting NEC-Associated Biomarkers

The advantage of metabolomics and proteomics as means of biomarker discovery in neonates and small infants lies in their ability to simultaneously quantify an extremely high number of small molecules using very small quantities of specimens. However, the high number of molecules detected is also a drawback, considering that the extensive datasets require a significant amount of work, computational processing, special bioinformatics software, and complex statistical approaches tailored to the specific molecules in order to detect associations and validate the pathways indicated by the discovered metabolites (108).

The multifactorial pathophysiology of NEC and common co-morbidities involving inflammation (e.g., sepsis and BPD) result in multiple confounding factors that are difficult to control for. Moreover, in the absence of reference values for metabolite and proteins detected by omics, biomarker detection utilizing an omics approach depends on comparison of patients with healthy controls. Selecting a strictly matched control group of completely healthy PTI may prove to be a hard task given the multiple endogenous and environmental factors associated with prematurity, even in the absence of prematurity - associated co-morbidities. Another limitation of metabolomics and proteomics evaluation is the lack of GA- and chronological age-specific reference values in different specimens leading to expression of results as values upregulated and downregulated in relation to the controls, a fact which makes comparisons among studies more challenging.

Additionally, the lack of a known omics-derived standard for confirmation of NEC diagnosis makes analyzing and interpreting the data even more difficult. In this sense, the capability of metabolomic and proteomic approaches to provide insight into NEC pathophysiology and define NEC-specific biomarkers is compromised. A way to overcome this limitation may be the employment of an integrative multiple-omics approach, which offers a more precise overview of the mechanisms underlying NEC development and outcome (121, 122). A multiple-omics approach has been employed by four of the 15 published studies dealing with metabolomics or proteomics in NEC, which integrated two-omic analyses (87, 105, 106, 109). The large amount of data derived from an integrative multiple-omics analysis requires appropriate statistical tools and a sufficiently large number of subjects. The latter requirement must be considered in the light of the difficulties in gathering sufficient numbers of NEC-patients and “healthy” matched controls. The small sample size constitutes a constant limitation of previous studies on NEC metabolomics and proteomics, which included a mean number of 27 infants with NEC (range 3–85) and 49 infants with sepsis/NEC (range 10–77), leading to underpowered results. Therefore, large-scale, multicenter studies with pre-determined sample size must be conducted to obtain results sufficiently powered. The development of a NEC Registry and Biorepository by the NEC Society will make a significant contribution in this direction by promoting international collaboration among institutions through the shared use of biologic samples (123, 124).

Another limitation regarding the population included in previous omics studies is the use of different criteria for defining NEC. The most commonly used definition was based on Bell's staging II–III (82, 87, 102, 109), or stages I–III (110, 111), clinical and radiological findings (105–107, 112), clinical symptoms and treatment with antibiotics (101), radiology and/or intestinal pathology findings (88), or criteria for NEC were not reported (96, 100, 104, 109). During the last decade, the value of Bell's criteria for NEC diagnosis has been debated due to their inability to discriminate NEC from other non-NEC systemic or intestinal diseases (8). An ideal definition of NEC must take into account the disease pathophysiology, demographics of the target population, and clinical presentation, and be capable of prediction, early diagnosis, and differentiation between medical and surgical NEC, as well as between preterm NEC and spontaneous intestinal perforation or other non-NEC intestinal diseases.

Finally, financial restraints limit the broader application of omics for the detection of NEC biomarkers considering that sophisticated and expensive equipment are required, which may not be available at all centers, along with skilled workforce, which render the processing of each sample very costly.

### Emerging Omics

Other omics emerging as promising for detection of biomarkers for NEC diagnosis include metagenomics, genomics, and transcriptomics.

Metagenomics is the study of genetic material recovered directly from environmental samples such as the gut. During the past decade, several studies have explored the microbial DNA signatures in the gut of term and preterm infants using either targeted (16S rRNA gene) or non-targeted (shotgun) metagenomics. Results of these studies have provided new insights into the role of the gut microbiome in the pathogenesis of NEC, sepsis, and other neonatal morbidities, as discussed in the pathogenesis section of the current review (21). The changes in preterm infants' microbiome that have been associated with NEC development could be further evaluated as predictive and diagnostic biomarkers (33, 35).

Genomics is the study of all genes in an organism. Epidemiological data suggesting genetic predisposition to NEC prompted investigators to study the genome of preterm infants targeting genes encoding factors involved in NEC pathogenesis (3, 44). Experimental and/or clinical studies revealed several gene variants that were associated with increased risk of NEC, including, but not limited to, variants in the genes encoding nucleotide binding and oligomerization domain (NOD)-like receptors (NLRs), autophagy-related protein (ATG), angiotensinogen (AGT), IL8, KNG1- low-molecular-weight kininogen (LMWK) protein, ACACB- Acetyl-CoA Carboxylase Beta, and CAT- Catalase, and genes regulating the pathways of the receptors TLR2, TLR4, and TREM1 (47, 48, 125–128). Thus, far, the accuracy of genomic biomarkers in NEC diagnosis has not been evaluated.

Transcriptomics is the study of the transcriptome, e.g., the study of the mRNA within a cell or organism. Experimental and clinical studies as well as a metanalysis on long-non-coding RNA (lncRNA), micro RNA (miRNA), and mRNA profiles in NEC demonstrated a role of lncRNA, miRNA, and mRNA in NEC and suggested further evaluation of certain lncRNA and miRNA as potential biomarkers in NEC (45, 129–133).

## Conclusions and Future Research

Modern technology has contributed to the discovery of numerous proteins and other products of metabolism that are capable of differentiating between NEC and non-NEC conditions, with some of them being able to predict NEC development days and weeks prior to the appearance of clinical manifestations. However, so far, there is no consensus among researchers for a distinct metabolomic/proteomic profile specific for NEC due to the significant inconsistency of findings among studies. Nevertheless, metabolomic and proteomic analysis using modern technology has opened new horizons in research for the discovery of novel biomarkers of NEC that could offer an important aid in prediction, early diagnosis, and prognosis. A first step to this goal is redefining NEC. Future research should (a) continue to explore for non-invasive, NEC-specific, single biomarkers or panels with high prognostic and diagnostic value; (b) simplify the assessment incorporating the candidate biomarkers into a single diagnostic test in collaboration with industry; and (c) propose diagnostic algorithms integrating clinical and laboratory metadata with multi-omics data.

Recent advances in high-throughput molecular technologies and other developments that could provide researchers with opportunities to accomplice these goals include (a) integration of multiple omics, such as metabolomics, proteomics, genomics, transpcriptomics, lipidomics, metagenomics, metascriptomics, epigenomics, and methylomics that may provide a more precise, in-depth overview of the biological processes underlying NEC development and progression; (b) the use of nanotechnology to build a multiplexed NEC magnetic nanoparticle-based biosensor platform of biomarkers detected by omics further broadens the prospect for discovery of rapid and inexpensive biomarker panels to be used at the bed-side (134); (c) the development of bioinformatics software which allows further analysis of complex data derived through omics technology, such as PANTHER bioinformatics software (Protein ANalysis THrough Evolutionary Relationships, University of Southern California, Los Angeles, CA, USA), Pathway Studio software (Ariadne Genomics, Rockville, MD, USA), and other relative tools. Such software constitutes a valuable tool for exploring molecular interactions between the cause and the biological processes and delineating the metabolic and protein networks and pathways underlying the development and progression of NEC (135); and (d) finally, the development of the Registry and Biobank by the NEC Society, which will provide opportunities for further collaborative research on NEC-specific biomarker discovery (123, 124).

## Author Contributions

EA and KS conceptualized the article. EA, CA, and HG wrote the article. KS and CA supervised the writing and critically revised the manuscript. All authors contributed to the article and approved the submitted version.

## Conflict of Interest

The authors declare that the research was conducted in the absence of any commercial or financial relationships that could be construed as a potential conflict of interest.

## Abbreviations

^1^H NMRS: proton nuclear magnetic resonance spectroscopy
AUC: area under the curve
BW: birth weight
GA: gestational age
GC–MS: gas chromatography – mass spectrometry
I-FABP: intestine-fatty acid binding protein
LC-MS: liquid chromatography-mass spectrometry
LOS: late onset sepsis
MS: Mass Spectrometry
NEC: necrotizing enterocolitis
SAA: serum amyloid-A
VLBWI: very low birth weight infants.
